# Single-cell transcriptomic analysis of canine insulinoma reveals distinct sub-populations of insulin-expressing cancer cells

**DOI:** 10.1186/s44356-025-00026-3

**Published:** 2025-05-26

**Authors:** M. D. Wallace, M. E. Herrtage, R. Gostelow, L. Owen, L. Rutherford, K. Hughes, A. Denyer, B. Catchpole, C. A. O’Callaghan, L. J. Davison

**Affiliations:** 1 Department of Clinical Science and Services, Royal Veterinary College, Hatfield, UK; 2 Department of Physiology, Anatomy and Genetics, University of Oxford, Oxford, UK; 3 Department of Clinical Veterinary Medicine, University of Cambridge, Cambridge, UK; 4 Department of Pathobiology and Population Sciences, The Royal Veterinary College, North Mymms, Hawkshead Lane, Hatfield, Herts AL9 7 TA UK; 5 Centre for Human Genetics, University of Oxford, Oxford, UK

**Keywords:** scRNA-seq, insulinoma, canine, Boxer, EGR1, P53

## Abstract

Canine malignant insulinoma is a rare, highly metastatic and life-threatening neuroendocrine tumour of pancreatic beta cells. To map the single-cell transcriptomic landscape of canine insulinoma for the first time, transcriptomic profiles of 5,532 cells were captured from two spontaneous insulinomas (Patient 1 and 2) and one associated metastasis (Patient 2) in two Boxer dogs. Distinct cancer, endocrine, and immune cell populations were identified. Notably, all three tumour samples contained two transcriptionally distinct insulin-expressing tumour cell populations (INS^+^ and INS^+^FOS^*low*^), characterised here for the first time. These two cancer cell populations significantly differed by ~ 8,000 differentially expressed genes (DEGs), particularly tumour suppressor genes (e.g. *TP53*, *EGR1*) and cancer-related pathways (e.g., MAPK, p53). In contrast, *COX7A2L* was one of a few genes ubiquitously expressed and significantly upregulated (> 20-fold) in both insulin-expressing tumour populations compared to other captured populations. Both populations were also characterised by expression of chromogranin/secretogranin neuroendocrine tumour marker genes (e.g. *CHGA*, *SCGN*). There were far fewer gene expression differences observed between insulin-expressing tumour cells from the two patients (~ 600 DEGs) than between the two cancer cell populations within each patient. These DEGs included *CLTRN*, *TMSB4X*, *CSRP2*, *LGALS2*, and *C15orf48.* Unexpectedly for a tumour of endocrine origin, the metastasis in Patient 2 exhibited > 20–70 fold upregulation of exocrine pancreatic genes including *CLPS*, *PRSS2*, *PRSS* and *CTRC*. Immune cell analyses identified distinct infiltrating immune populations, including memory T cells and macrophages and revealed likely tumour-immune interactions, including the CD40-CD40L interaction. This study provides the first single-cell RNA sequencing (scRNA-seq) analysis of naturally occurring insulinoma in any species, revealing tumour cell heterogeneity, novel immune microenvironment features, and potential therapeutic targets. Despite its small scale, the findings highlight the utility of scRNA-seq in veterinary oncology and its translational potential for pancreatic neuroendocrine tumours across species.

**Supplementary Information:**

The online version contains supplementary material available at 10.1186/s44356-025-00026-3.

## Introduction

Canine malignant insulinoma is a rare, but life-threatening, functional neuroendocrine tumour of pancreatic beta cells [5], also known as an islet cell carcinoma. Typically, middle-aged to older dogs are affected, with certain breeds including the Boxer, German shepherd dog, Labrador retriever and West Highland white terrier predisposed [34]. Clinical signs may include increased appetite, weight gain, and weakness between meals or after exercise. This may eventually progress to collapse and hypoglycaemic seizures. Diagnosis is usually based on documentation of clinical signs and inappropriately high serum insulin concentration in the presence of concurrent hypoglycaemia. Diagnostic imaging may also support the diagnosis, since in many cases a tumour nodule may be present visible in the pancreas by ultrasound or cross-sectional imaging [6]. There is some overlap in histopathological phenotype between canine insulinoma and human pancreatic neuroendocrine tumours [13], although a benign non-invasive insulinoma phenotype has been observed in humans [14].

Treatment for insulinoma is based on surgical excision where possible, but this is rarely curative [11]. The high metastatic potential of the tumour means that the prognosis remains poor, since functional metastases often lead to later recurrence of clinical signs. Adjunctive medical therapy is usually aimed at mitigating clinical signs by increasing hepatic gluconeogenesis, antagonising insulin effects or reducing insulin release, using drugs such as prednisolone, octreotide or diazoxide. Potential benefits of drugs that are toxic to the beta cells (e.g. streptozotocin) and anti-cancer tyrosine kinase drugs (e.g. toceranib) have been reported in small studies, but they carry risk of side effects and their efficacy is currently unclear [30, 36].

The largest study of canine insulinoma published to date provided survival data on 93 dogs that underwent medical and/or surgical treatment and reported a median survival of 8 months (range 3 to 16 months) with medical management and 20 months (range 9 to 43 months) with surgery [34]. There is therefore a clear unmet need for novel, targeted treatments in canine insulinoma. In addition, tumour biomarkers would be valuable in helping to predict which patients are at risk of metastasis and would benefit from additional therapy post-surgery.

**Fig. 1:**
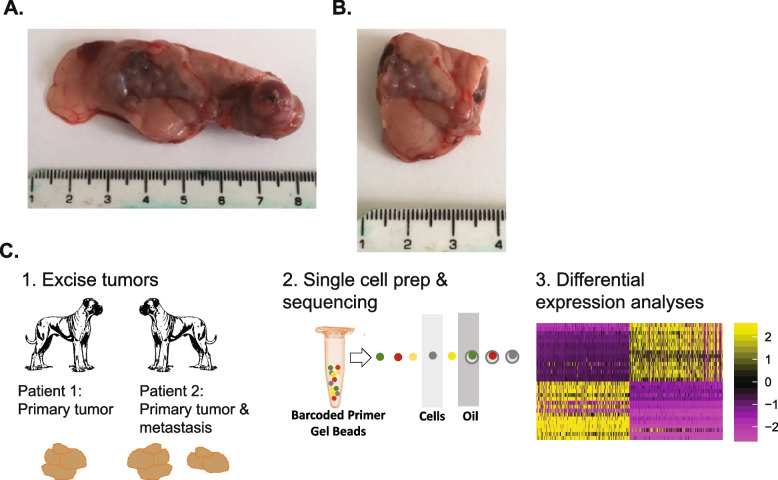
Canine insulinoma study overview. **A** Primary tumour tissue from an 8-year-old female neutered Boxer diagnosed with insulinoma (Patient 2) received after tissue had been removed for diagnostic purposes. **B** Metastatic lesion tissue from Patient 2 received after tissue had been removed for diagnostic purposes. **C** Study overview depicting excision, cell preparation, scRNA-seq, and bioinformatic analyses of tumour samples from Boxers diagnosed with insulinoma

Single cell transcriptomics provide an unparalleled opportunity to explore the biology of tumours in more detail from gene expression profiles of individual cells [40]. Sequencing single cells overcomes the difficulties inherent in bulk RNA-sequencing of heterogeneous cell populations. Single cell RNA-sequencing (scRNA-seq) helps to dissect pathological processes, to identify unique features of malignant cells, and to study the cellular composition of the tumour and the tumour microenvironment (TME) [29]. To date, only human and mouse pancreatic neuroendocrine tumours have been examined using single cell transcriptomics [17]. Canine osteosarcoma has been examined utilising scRNA-seq [3] but not canine insulinoma. Here we present single cell RNA profiles of 5,532 individual cells from fresh canine insulinoma tissue (*n* = 2 tumours and *n* = 1 metastatic lesion) which allow us to generate new hypotheses about driving factors and potential treatment targets (Fig. 1).

## Materials and methods

### Tissue samples

Excised primary tumour from two adult female neutered (unrelated) Boxer dogs diagnosed with pancreatic islet cell neoplasms consistent with insulinoma were included in this study (Supplementary Table 1), as well as metastatic tissue from Patient 2. Insulinoma tissue was surplus to diagnostic requirements for histopathology following planned therapeutic surgical tumour excision during laparotomy under general anaesthesia at either the Queen Mother Hospital for Animals, Royal Veterinary College or the Queen’s Veterinary School Hospital, University of Cambridge. The diagnosis, decision to proceed to surgery, and the surgical plan were determined by the clinician in charge of the case in conjunction with the owner and were not influenced by the study. Owner consent was given for use of surplus tissue for scRNA-seq. In both cases, the diagnosis of pancreatic islet cell neoplasm, consistent with insulinoma given the clinical context, was confirmed by histopathological evaluation of excised tissue. In both dogs, blood glucose normalised following surgical excision of the insulinoma. Both patients were alive and not requiring any further medical therapy at 12 months post-surgery, but no further follow up information is available.

Patient 1 was a 9-year-old female neutered Boxer, diagnosed with insulinoma based on a 6-week history of progressive lethargy and occasional collapse at exercise, with hypoglycaemia and inappropriately elevated serum insulin concentration. A single small pancreatic nodule (10 mm × 15 mm) was evident on pancreatic ultrasound, subsequently confirmed as an iso-attenuating nodule slightly deforming the margins of the pancreas on abdominal CT scan.

Patient 2 was an 8-year-old female neutered Boxer, diagnosed with insulinoma based on progressive collapsing episodes over several weeks, hypoglycaemia and inappropriately elevated serum insulin. A large multi-nodular primary nodule (70 mm × 25 mm x 10 mm) was noted on pancreatic ultrasound. In addition, a solitary gross metastatic lesion in the abdomen, attached to the peritoneum, was observed at surgery and removed at the same time as the primary tumour. This patient also suffered from a hypoglycaemic seizure the day prior to surgery.

#### Sample processing

After diagnostic samples were completed, all visible exocrine pancreas was trimmed from the tissue, the sample was placed into fresh cold phosphate-buffered saline containing 10% foetal calf serum (Gibco) for transport to the laboratory on cold packs. Following arrival in the laboratory (within 2 h of initial surgical excision), the sample was processed into a single cell suspension. In the case where a metastatic lesion was available, this was processed in parallel, in a separate tube. Briefly, blunt dissection was performed using sterile scissors to cut the sample into small pieces of < 1 mm diameter. Following a brief centrifugation step, the supernatant was discarded, and sample resuspended in 5 ml of Liberase TL (Roche) in Hanks’ Balanced Salt Solution (HBBS – Sigma), followed by incubation for 13–14 min at 37 degrees, shaking every few minutes. The tube was then transferred to ice, and 10 ml of HBBS with 0.2% Bovine Serum Albumin (BSA—Sigma) added, followed by vigorous shaking for 30 s to 1 min. The sample was allowed to stand on ice for 5 min and the supernatant carefully removed with a pipette, leaving a residual volume of cells and media of 2–3 ml. An additional 12–13 ml of HBBS/2% BSA was added, the tube inverted and replaced onto ice for 5 min. This wash step was repeated twice, with the final wash in HBBS alone. The cells were then centrifuged at 1000 g for 5 min and the supernatant carefully removed by pipetting. The cells were resuspended in an appropriate volume (of approximately 5 × the pellet volume, up to 500ul) of pre-warmed TrypLE (Gibco) and the cells mixed by gentle pipetting, before incubation for 11 min. The reaction was terminated by adding RPMI Medium without glucose (Gibco) with 10% Foetal bovine serum (FBS—Sigma) at a volume 5x that of the TrypLE added. The samples were centrifuged at 1000 g for 5 min and supernatant removed by pipetting before resuspension in 500ul RMPI/FBS. The TrypLE step was repeated if more clumps than single cells were visible on microscopy, and cell viability was assessed using Trypan Blue. The final sample was passed through a 40 um cell strainer.

Red cell lysis, further viability assessment, sample partitioning and library preparation were undertaken by the Oxford Genomics Centre using the 10X Chromium system. Based on cell count and live-dead staining, an estimated 1000–2000 live cells per sample were utilised for library preparation from each sample. Single-cell 3’ gene expression libraries were prepared for each sample using a Chromium Single Cell 3 v2 Kit and dual index library construction kit (10x Genomics). Sequencing was performed on a HiSeq4000 sequencer (Illumina) across 2 lanes in total.

### scRNA-seq data analyses

Raw scRNA-seq reads were processed and aligned to CanFam3.1 by Cell Ranger (3.1.0) followed by QC, cell clustering, differential expression analyses, and visualization in R using Seurat (3.1.2 and 5.1.0) [7, 37]. Transcriptomic profiles of 5,532 cells were captured with an average of 123,536 reads per cell and transcripts from a median of 1,010 genes detected per cell. For QC, cells were required to have > 50 features and < 10% mitochondrial read content. The cell type corresponding to each cluster was determined through standard marker expression, top distinguishing markers, and automatic classifiers (scMCA 0.2.0, scATOMIC 2.0.3) [29]. Cell cycle phase was also classified by scATOMIC. In differential expression analyses, Seurat adjusts p-values (adj.p) based on Bonferroni correction using all features in the dataset.

Over-representation KEGG pathway analyses were conducted on DEGs having adjusted *p*-values < 0.05 and > twofold change, using clusterProfiler (4.10.1) [45, 46, 49] and org.Cf.eg.db (3.18.0). The stemness score was calculated with TCGAbiolinks (2.30.4) [12, 25]. Copy number alteration (CNA) analysis was conducted with InferCNV (1.18.1) using the average expression across all cells from the RNA counts matrix and the 0.1 cutoff recommended for 10 × data [39]. CellChat (2.1.2) was used to conduct the cell–cell communication analysis [19, 20].

The scRNA-seq reads have been deposited in the European Nucleotide Archive (ENA project: PRJEB86016/study ERP169389; samples: ERS23781655, ERS23781656, and ERS23781657).

## Results

**Fig. 2:**
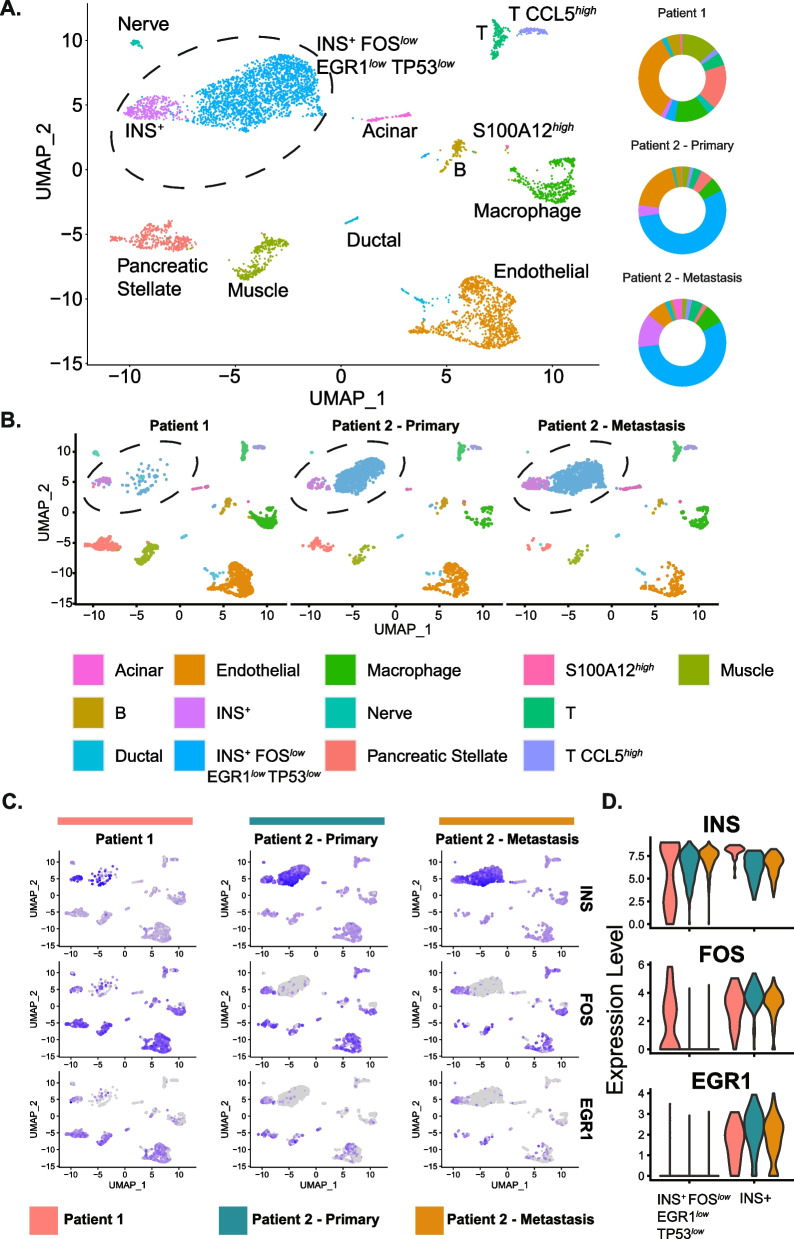
Cell populations captured from scRNA-seq of canine insulinoma samples. **A** (Left) UMAP showing cells captured from tumours. The cell type corresponding to each cluster of cells was determined through standard marker expression, top distinguishing markers, and automatic classifiers (scMCA 0.2.0, scATOMIC 2.0.3). (Right) Doughnut plots showing the proportions of each cell type captured from each tumour sample. See Supplementary Table 3 for details. **B** UMAPs showing levels of select standard marker or distinguishing marker genes for cell type classification: Acinar (CEL), Alpha cell (GCG), B cell (MS4A1), Delta cell (SST), Ductal cell (KRT19), Endothelial cell (PLVAP), Gamma cell (PPY), *INS*^+^ *FOS*^*low*^ (INS, FOS, EGR1), *INS*^+^ (INS), Macrophage (CD86), S100A12^*high*^ (CD86, S100A12), Nerve (PLP1), Pancreatic stellate (COL1A2), T (CD3E), T CCL5^*high*^ (CD3E, CCL5), Muscle (ACTG2). See Supplementary Table 4 for cluster distinguishing markers and Supplementary Table 10 for scATOMIC classification

To interrogate the transcriptomic landscape, heterogeneity, and tumour microenvironment of cell populations in canine insulinoma, single cell RNA-sequencing (10x Genomics) was conducted on two primary insulinoma tumours and one metastasis from two unrelated Boxer dogs diagnosed with insulinoma and undergoing therapeutic surgical excision (Fig. 1, Supplementary Tables 1–2). Transcriptomic profiles of 5,532 cells were captured, and scRNA-seq analyses revealed distinct cancer, endocrine, and immune cell populations (Fig. 2, Supplementary Tables 3–5). These included insulin-expressing cancer cells, acinar cells, B cells, ductal cells, macrophages, T cells, muscle, nerve, and pancreatic stellate cells. No normal beta, alpha, or delta cell clusters were identified, consistent with these cell types comprising only a small proportion of cells normally present in the pancreas and consistent with the cells being from tumour masses rather than purified islets.

### Characterization of two distinct insulin-expressing tumour cell populations

**Fig. 3:**
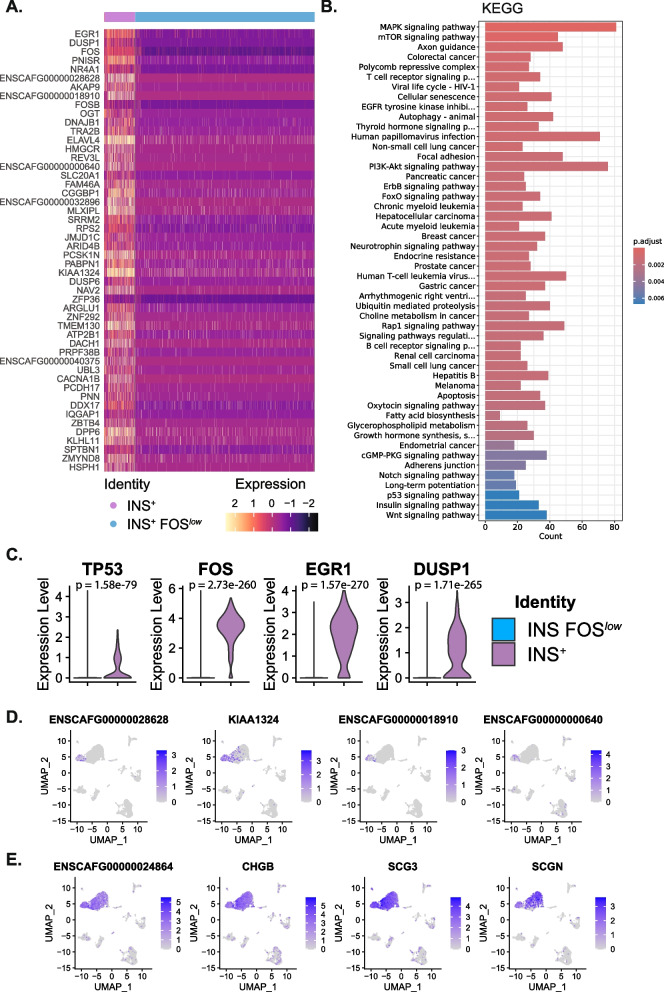
Characterization of two distinct insulin-expressing cancer cell populations found within each tumour. **A** Heatmap of the top 50 DEGs between the *INS*^+^ *FOS*^*low*^ and *INS*^+^ clusters (adj.*p* <.05, avg log2 FC > 1). **B** KEGG over-representation pathway analysis of the DEGs between the *INS*^+^ FOS^*low*^ and *INS*^+^ clusters (adj.*p* <.05). **C** Violin plots of *TP53, FOS, EGR1,* and *DUSP1* expression between the insulin-expressing clusters, showing significant downregulation in the *INS*^+^ *FOS*^*low*^ cluster. **D** UMAP plots showing downregulation of select DEGs in the *INS*^+^ cluster. **E** UMAP plots showing expression of chromogranin and secretogranin family genes in both the *INS*^+^ *FOS*^*low*^ and *INS*^+^ clusters

Notably, two distinct insulin-expressing cancer cell populations were captured in all three samples (Fig. 2A). Differential expression analyses showed these two insulin-expressing populations had distinct transcriptomic profiles from each other with ~ 8,000 differentially expressed genes (DEGs) (Fig. 3, Supplementary Table 6). The two populations could be distinguished by expression of *FOS* (13.1 fold-change (FC)), *EGR1* (14.4 FC), and *TP53* (2.3 FC).

The *INS*^+^ *FOS*^*low*^ *EGR1*^*low*^ *TP53*^*low*^ (abbreviated INS^+^ *FOS*^*low*^) population was characterized by strong downregulation of many genes compared with the *INS*^+^ *FOS*^+^ *EGR1*^+^ *TP53*^+^ (abbreviated *INS*^+^) population and all other cell populations identified (Fig. 3A, C, Supplementary Tables 6–7). In addition to *TP53* and *EGR1*, ~ 60 other tumour suppressor genes were expressed at significantly lower levels in the *INS*^+^ *FOS*^*low*^ population, compared to the *INS*^+^ population (adj.*p* < 0.05, log2 FC < − 1, min.pct 0.1) (Table 1, Supplementary Table 6).

**Table 1:**
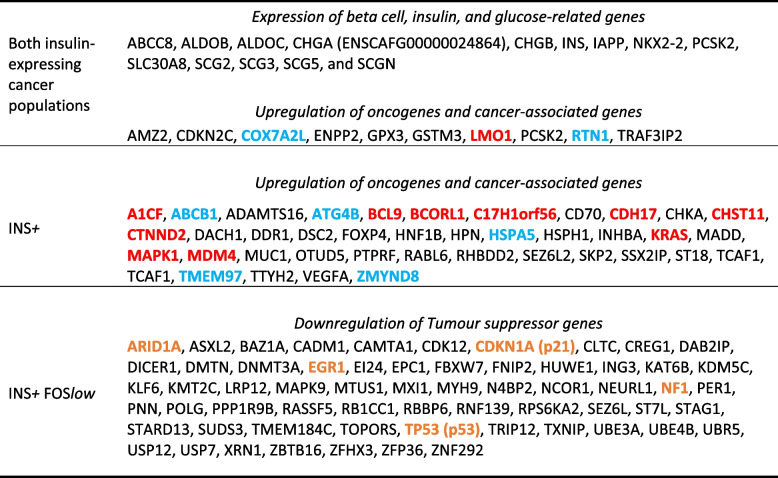
Expression landscape in insulin-expressing cancer cells of canine insulinoma

Note, not all DEGs are shown here. See Supplementary Data for the full analyses. Selected DEGs shown are Bonferroni-adjusted *p*-values < 0.05 and demonstrated at least two-fold change. UniProt, RefSeq, and COSMIC were mined for gene classification. Oncogenes are coloured red. Genes previously highlighted for their therapeutic potential in cancer literature are coloured blue. Particularly striking tumour suppressor genes frequently deregulated in cancer are highlighted orange

Several DEGs distinguished the *INS*^+^ cluster from the *INS*^+^ FOS^*low*^ cluster and other cell populations. These included *ENSCAFG00000028628* (TMEM178B), *KIAA1324* (ELAPOR1), *ENSCAFG00000018910*, *ENSCAFG00000000640*, *ENSCAFG00000037190*, and *ENSCAFG00000040375* (Fig. 3D, Supplementary Tables 4–6).

Comparing the DEGs from one insulin-expressing cluster to the other, KEGG pathway analysis showed an enrichment of genes in several pathways well-known for their deregulation in cancer, such as MAPK, mTOR, PI3K-AKT, Notch, Wnt, and p53 signalling pathways as well as cellular senescence and apoptosis pathways (Supplementary Table 7, Fig. 3B).

Despite their many differences, the two insulin-expressing cancer cell populations shared strong expression of other beta cell and glucose/insulin-related genes (such as *IAPP*, *PCSK2*, *NKX2-2*, *ABCC8*, *ALDOB*, *ALDOC*, and *SLC30A8*) as well as chromogranin and secretogranin family genes (*CHGA* (*ENSCAFG00000024864*), *CHGB*, *SCG2*, *SCG3*, *SCG5*, and *SCGN*) (Fig. 3E, Supplementary Tables 4–5). *COX7A2L* was one of very few genes that showed both ubiquitous expression and significant upregulation in both insulin-expressing populations compared to the cells in other clusters (adj.*p* = 0, log2 FC = 4.76).

Insulin-expressing cells, particularly the *INS*^+^ cell cluster, also showed higher expression of specific oncogenes and cancer-related genes, including genes that have been prioritized for targeted therapy in humans (Table 1, Supplementary Table 4).

Copy number analysis revealed there were more copy number alterations (CNAs) in the *INS*^+^ FOS^*low*^ population compared to the *INS*^+^ population (Supplementary Fig. 2). In Patient 2, who exhibited more advanced disease, the *INS*^+^ *FOS*^*low*^ cells showed frequent deletion of chromosomes 13, 20, & 29 and amplification of 11, 14, & 15. However, these CNAs were less frequent in the *INS*^+^ cells of Patient 2 and were not frequently altered in either insulin-expressing population in Patient 1. Moreover, CNAs in Patient 1, who exhibited less advanced disease, were only detected in small subsets of cells. Together, this suggested that whole-chromosome CNAs were not an early driving initiator of these insulinomas, rather they may play a more important role later in the evolution of the tumour.

### Heterogeneity between the primary tumours

Cross sectional imaging, with computed tomography demonstrated a single small pancreatic mass in Patient 1. There was no evidence of metastasis, and the patient had relatively mild clinical signs, indicating a less advanced disease stage. The insulinoma in Patient 1 contained a considerably lower proportion of insulin-expressing cells than the primary tumour in Patient 2 (a total of 5.5% versus 59.5%, Fisher's Exact *p* < 2.2e- 16) (Supplementary Table 3, Fig. 2A). However, a greater proportion of those cells were in the G2/M phase of the cell cycle (Fisher’s Exact, *INS*^+^ *FOS*^*low*^ *p* = 2.85e- 06, *INS*^+^ *p* = 0.0074, Supplementary Fig. 3).

Patient 2 had a larger pancreatic mass with evidence of suspected metastasis due to a second mass lesion present within the peritoneum, consistent with more advanced disease. In Patient 2, most cell populations were present in similar proportions in both the primary tumour and the metastatic lesion, with 69.0% of cells in the metastasis expressing insulin, and an increase in proportion of cells in the *INS*^+^ cluster compared to *INS*^+^ *FOS*^*low*^. The absence of any additional immune cell populations suggested locally invasive metastasis rather than spread of the tumour to a draining lymph node (Fig. 2A, Supplementary Table 3). Rapidly dividing cancer cells are often found in S phase, making DNA replication a target for many cancer therapies. Consistent with this, across the different cell types examined, the insulin-expressing tumour cells had the greatest proportion of cells in S phase, with the highest being a population of insulin-expressing cells in the metastasis.

**Fig. 4:**
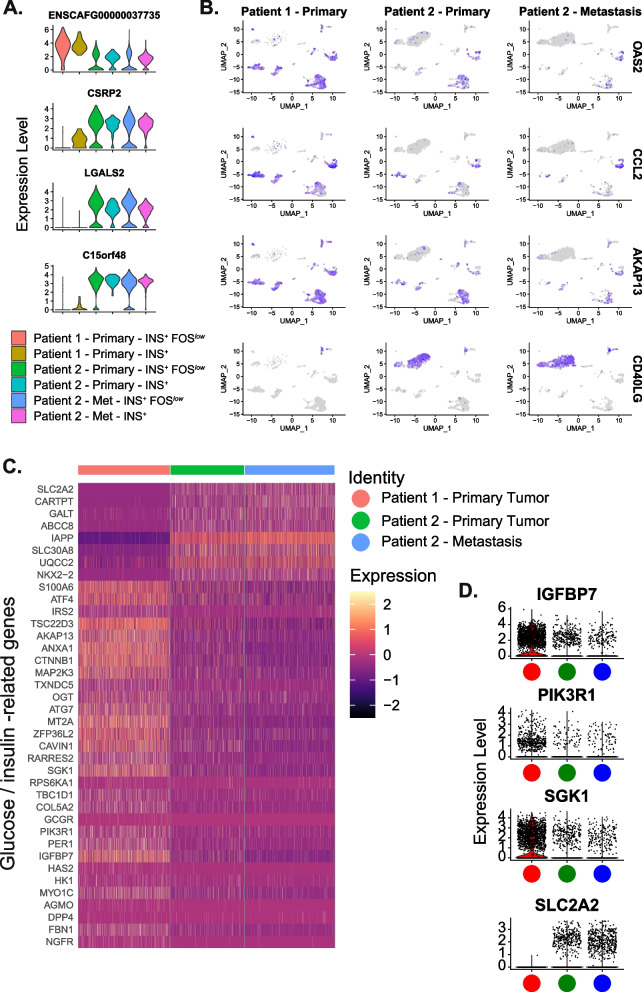
Heterogeneity between primary tumours. **A** Violin plots of select genes that were differentially expressed in both insulin-expressing populations when comparing the primary tumours of Patient 1 to the primary tumour of Patient 2. **B** UMAP plots showing upregulation of immune/inflammatory-related genes *OAS2*, *CCL2*, and *AKAP13* in Patient 1 compared to Patient 2 across the cell populations captured and upregulation of *CD40LG* in Patient 2 in both populations of insulin-expressing cancer cells, compared to these cells in Patient 1. **C** Heatmap of beta cell, glucose, and insulin-related DEGs between the primary tumours. Expression in the metastasis of Patient 2 is also shown for comparison. **D** Violin plots of select glucose and insulin-related DEGs from Fig. 4 C: *SLC2A2*, *PIK3R1*, *IGFBP7*, and *SGK1* across samples – Patient 1 (red), Patient 2 primary (green), Patient 2 metastasis (blue)

Genes that were differentially expressed between cancer cells from the two primary tumours included upregulation of *ENSCAFG00000037735* (*TMSB4X*) and downregulation of *CSRP2*, *LGALS2*, and *C15orf48* in Patient 1 compared to Patient 2 (Fig. 4A, Supplementary Table 8). *CLTRN*, a stimulator of beta cell replication, was upregulated in both the primary tumour and metastasis of Patient 2 compared to the primary tumour of Patient 1.

Pathway analyses indicated a difference in immune/inflammatory related signatures between the two primary tumours, such as an enrichment of DEGs in the NF-kappa B signalling and TNF signalling pathways (Supplementary Table 9, Supplementary Fig. 4). Examples of genes driving these observed pathway differences include the strikingly greater expression of *OAS2*, *CCL2*, and *AKAP13* in Patient 1 compared to Patient 2 across several cell populations (Fig. 4B). In contrast, Patient 2 showed much higher expression of *CD40LG* in both populations of insulin-expressing cancer cells, compared to these cells in Patient 1 (Fig. 4B).

There were also expression differences between the two primary tumours in ~ 50 insulin/glucose-related genes, such as *SLC2A2*, which encodes the GLUT2 glucose transporter and *PIK3R1,* involved in the metabolic actions of insulin (Fig. 4C, D).

### The insulinoma metastasis is characterized by upregulation of exocrine pancreatic genes

It is important to understand the biology of metastasis in canine insulinoma because the main reason for the poor long-term prognosis is the development of functional insulin-secreting metastatic lesions after surgery. Therefore, a differential expression analysis was conducted comparing the metastasis to the primary tumour in Patient 2.

**Fig. 5:**
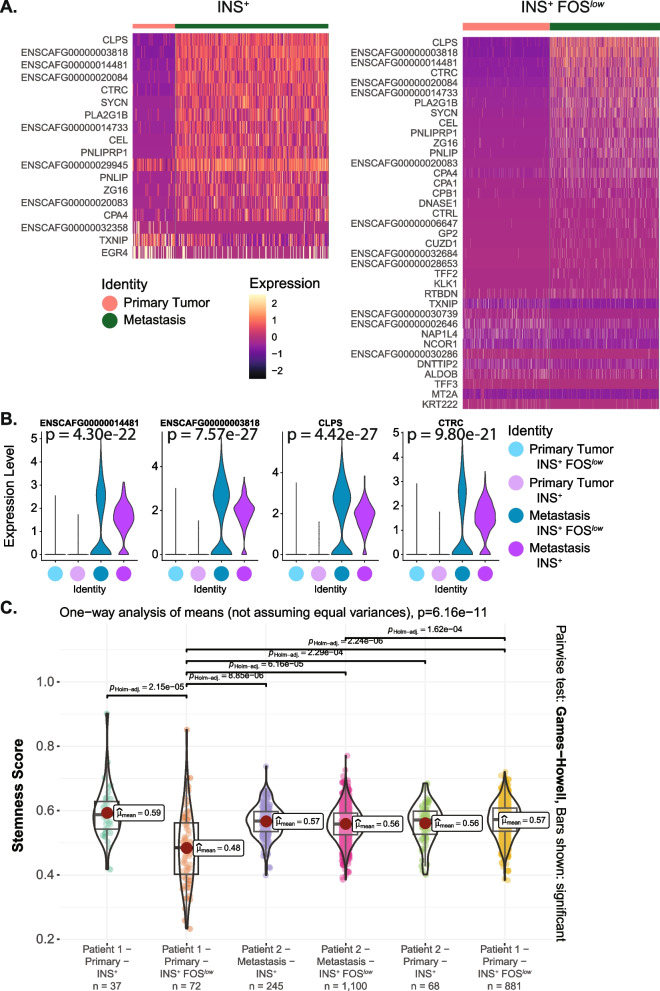
Differentially expressed genes in metastatic insulin-expressing tumour cells compared to primary tumour in canine insulinoma. A Heatmap of *INS*^+^ cluster (left) and *INS*^+^ *FOS*^*low*^ cluster (right) DEGs between primary tumour and metastasis (adj.*p* <.05, avg log2 FC > 1). **B** Violin plots showing upregulation of pancreatitis markers in both insulin-expressing cancer cell populations in the metastasis compared to primary tumour. P-values shown reflect the analysis between the primary tumour and metastasis for the *INS*^+^ cell cluster. For the *INS*^+^ *FOS*^*low*^ cluster analysis and p-values, see Supplementary Table 11. **C** Violin plot showing that the insulin-expressing cancer cell populations have significantly higher stemness scores compared to the *INS*^+^ *FOS*^*low*^ population in the primary tumour of Patient 1. Shown are Holm-adjusted p-values from Games-Howell pairwise tests

Notably, the metastasis showed significant upregulation of several exocrine pancreatic marker genes including *CLPS*, *ENSCAFG00000014481* (*PRSS2*), *ENSCAFG00000003818* (*PRSS*), and *CTRC* (Fig. 5A, B).

Since cancer progression can involve the acquisition of stem cell-like features that make tumours more likely to spread to distant organs, more resistant to treatment, and result in poor prognosis, a ‘stemness’ score was calculated for the insulin-expressing cancer cells. The *INS*^+^ population in Patient 1 showed a significantly higher stemness score than the *INS*^+^ FOS^*low*^ cells (Fig. 5C, Supplementary Table 8). This elevated stemness score in the *INS*^+^ population of Patient 1 was similar to the stemness scores in the insulin-expressing cells of both the primary tumour and the metastatic lesion in Patient 2.

### Characterization of infiltrating immune cells in the tumour microenvironment

Single-cell transcriptomics provides an opportunity to characterise tumour-infiltrating immune cells, which may in turn provide insights into immune mechanisms of tumour control and so help to identify novel potential therapeutic targets. Here, the two CD3-expressing T lymphocyte clusters showed differential expression of the chemokines *CCL5* and *CCL4* (Supplementary Table 4). Classification of these cells using the scATOMIC tumour microenvironment cell type classifier [29], indicated that the T *CCL5*^*high*^ population consists primarily of Effector/Memory T cells (77/103), while the other T cell cluster consists predominantly of Naïve CD4+ T cells (152/223) (Supplementary Table 10). However this must be interpreted with caution as scATOMIC was trained on human cells and is not validated for canine data.

Both T cell populations showed strong expression of several inflammatory and interferon-related genes, such as *ENSCAFG00000006485* (interferon induced transmembrane protein IFITM), *CXCR4*, and *CLEC2D*. Consistent with this, the insulin-expressing cancer cells showed significant upregulation of inflammatory response genes, particularly *C15orf48* (NMES1) (adj. *p* = 0, log2 FC = 4.07) and *TRAF3IP2* (adj. *p* = 2.03e- 160, log2 FC = 4.03).

The other immune cell clusters identified were B lymphocytes, distinguished by CD19 expression, a population of mononuclear cells distinguished by high expression of *MS4A7*—a marker typically associated with an immunomodulatory macrophage phenotype, and a second population of mononuclear cells distinguished by high expression of *S100A12* – a marker of monocytes and early classically activated macrophages.

**Fig. 6:**
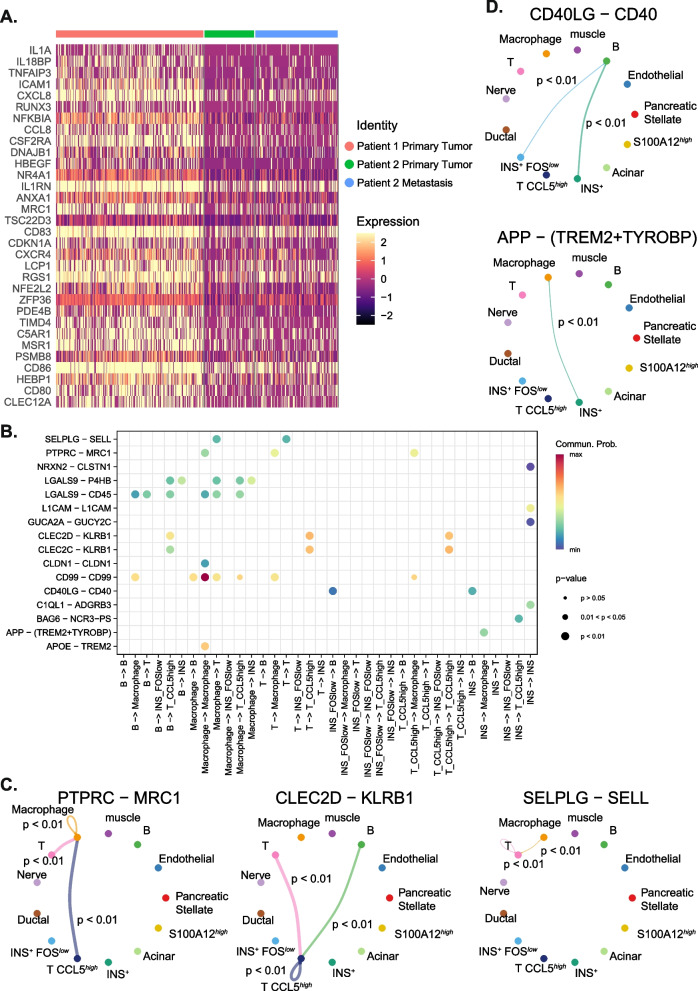
Characterisation of tumour-infiltrating immune cells and cancer cell response in canine insulinoma. **A** Heterogeneity of macrophage immune response between patients is shown in a heatmap of a subset of DEGs involved in immune-related processes. **B** Cell communication analysis showing significant interactions between insulin-expressing cancer cells and tumour-infiltrating immune cells. **C** Circle plots showing significant interactions of PTPRC—MRC1, CLEC2D—KLRB1, and SELPLG – SELL between immune cell populations. **D** Circle plots showing significant interactions of CD40LG − CD40 and APP − TREM2 + TYROBP between insulin-expressing cancer cells and immune cells

Comparing gene expression in the immune cell infiltrate between the two primary tumours, the greatest number of DEGs was seen in the macrophage (*CD86* and *MS4A7*-expressing) cluster (Fig. 6A, Supplementary Table 12). Pathway analysis showed an enrichment of DEGs involved in cytokine signalling (Supplementary Table 9, Supplementary Fig. 5). Fewer DEGs were found when comparing the immune populations between the primary mass and the metastatic lesion in Patient 2 (Supplementary Table 13).

Cell communication analysis showed significant potential for interactions between immune cells. Notable interactions included *PTPRC* (T cell, macrophage)—*MRC1* (macrophage) required for T-cell activation and TCR receptor signaling, *CLEC2D* (B cell, T cell)—*KLRB1* (T cell) associated with interferon gamma release, and *SELPLG* (macrophage, T cell) – *SELL* (T cell) important for immune cell trafficking to sites of inflammation (Fig. 6 B, C, Supplementary Table 14).

There were also significant interactions between insulin-expressing cancer cells and immune cells, namely *CD40LG* (*INS*^+^ *FOS*^*low*^, *INS*^+^) − *CD40* (B cell) and *APP* (*INS*^+^) − TREM2 + TYROBP (macrophage) (Fig. 6 B, D, Supplementary Table 14).

## Discussion

Canine insulinoma is an insulin-producing pancreatic neuroendocrine tumour with a poor prognosis. Studying the single-cell transcriptome of the primary and metastatic tumour has potential to reveal novel therapeutic targets, which might also be applicable to pancreatic neuroendocrine tumours in other species. Bulk RNA-seq of human and canine insulinoma and bulk ATAC-seq of human insulinoma has already provided valuable insights into the global regulatory, mutational and transcriptomic features of insulinoma [8, 33], but here we apply scRNA-seq analysis to canine insulinoma and identify two distinct subtypes of insulin-expressing cells within canine insulinoma for the first time.

Single cell transcriptomic analysis of human pancreatic neuroendocrine tumours has previously been reported, but no studies to date have included functional insulin-producing tumours. Single cell RNA-Seq of pancreatic neuroendocrine tumours in mice used tissue from an experimentally-induced model, rather than spontaneous disease [8, 17]. We provide the first scRNA-seq analysis of naturally occurring insulinoma in any species.

Three striking and unexpected findings emerged from this study. Firstly, all three insulinoma samples contained two distinct populations of insulin-expressing cancer cells, with very different transcriptomic profiles. Secondly, the metastatic insulinoma lesion, found in the abdomen attached to the peritoneum, contained all the different cell types represented in the primary tumour, rather than presenting as a single population of neoplastic insulin-expressing endocrine cells. Thirdly, the two insulin-expressing cell populations within the metastasis expressed high levels of exocrine pancreatic genes (e.g. *CLPS*, *PRSS2*, *PRSS*, and *CTRC*), which is atypical for a tissue of endocrine origin.

The two insulin-expressing clusters that were identified within each canine tumour can be described by their strong differences in transcription factor expression. They were the *INS*^+^ *FOS*^+^ *EGR1*^+^ *TP53*^+^ (abbreviated *INS*^+^), and *INS*^+^ *FOS*^*low*^ *EGR1*^*low*^ *TP53*^*low*^ (abbreviated *INS*^+^ *FOS*^*low*^) populations that demonstrated more than 8,000 differentially expressed genes (DEGs).

c-FOS is a transcription factor that forms part of the AP-1 complex and plays an important role in cell proliferation, differentiation, DNA repair and apoptosis [48]*.* The role of c-FOS varies by cancer type. In several types of cancer, high c-FOS is oncogenic and promotes tumour growth, cancer cell stemness and metastasis, and is a marker of poor overall survival [24, 42]. However, in some tumours, such as ovarian cancer, high c-FOS is associated with tumour suppressor activity and improved prognosis [31].

The *INS*^+^ *FOS*^*low*^ population was characterized by downregulation of key tumour suppressor genes including *TP53*, *EGR1, NF1, ARID1 A,* and *CDKN1 A* (p21). Loss of *TP53* and *EGR1* expression in the *INS*^+^ *FOS*^*low*^ population is notable given they are highly expressed and relatively ubiquitous in all the other cell populations analysed, including the *INS*^+^ population. *TP53* is the most frequently mutated gene in cancer and responds to diverse cellular stresses to regulate expression of target genes, thereby inducing cell cycle arrest, apoptosis, senescence, DNA repair, or changes in metabolism. EGR1 is a well-recognised transcription factor and tumour suppressor that activates p53, playing a role in the regulation of cell survival, proliferation, and cell death [41]. EGR1 is induced in insulinoma cells and pancreatic β-cells following stimulation with glucose [38], and loss of Egr-1 in mice also appears to predispose islets to ER (endoplasmic reticulum) stress and apoptosis [10].

*DUSP1* was one of the strongest downregulated genes in the *INS*^+^ *FOS*^*low*^ population compared to the *INS*^+^ population (23.5 fold-change). *DUSP1* has a complex and well-recognised role in cancer, as a regulator of MAPK, autophagy, cell proliferation and differentiation [35]. In contrast, the *INS*^+^ population maintains expression of *DUSP1*, but has heterogeneous upregulation of additional oncogenes, including *KRAS* and *MAPK1* compared to the *INS*^+^ *FOS*^*low*^ cells. Consistent with these specific findings, pathway analysis showed an enrichment of DEGs in several pathways well-known for their deregulation in cancer, such as MAPK and p53 signalling pathways.

Both insulin-expressing cancer cell populations expressed beta cell, insulin, and glucose-related genes, including known neuroendocrine genes such as chromogranins, secretogranins, and *IAPP* (islet amyloid polypeptide). In humans, chromogranins, particularly chromogranin A and *SCGN*, are associated with secretory granules in neuroendocrine cells, and are biomarkers of neuroendocrine tumours and a variety of diseases [4, 28].

Importantly, since both insulin-expressing populations were present in both the primary tumour and the metastasis, any novel therapeutic strategy in canine insulinoma would need to be effective at targeting both types of insulin-expressing cells. However, given the small scale of this study and the different clinical presentation of the two dogs, it cannot be assumed that the findings here will apply to all canine insulinomas. Nonetheless, when considering genes that may encode novel therapeutic targets of both tumour insulin-expressing cell populations, the small number of genes ubiquitously expressed and upregulated in both clusters included *COX7A2L* (also known as *COX7RP* and *SCAF1*), with a mean 27-fold upregulation. The encoded protein is a subunit of cytochrome c oxidase involved in mitochondrial respiration and electron transport. *COX7A2L* has also been associated with a range of cancers, including pancreatic, ovarian and breast cancer, with overexpression being a poor prognostic indicator [2, 23, 27]. Knockdown of *COX7A2L* reduces tumour growth in mice as well as proliferation in cancer cell lines, leading it to be proposed as an important therapeutic target [15, 18]. Therefore, this gene may warrant further investigation in canine insulinoma.

There were fewer differences between primary tumours in insulin-expressing cells (~ 600 DEGs) with only ~ 40 DEGs in the *INS*^+^ population between the two patients. Of these, some were differentially expressed in both insulin-expressing populations, including *ENSCAFG00000037735* (*TMSB4X*) and *CSRP2*. The functions of these genes in this context are not clear, but *CSRP2* has been associated with cancer cell stemness [51] and *TMSB4X* has been proposed as a cancer prognostic marker [47]. Of potential relevance to the ongoing use of the tyrosine kinase inhibitor toceranib in canine insulinoma, *TMSB4X* is also reportedly overexpressed in toceranib-resistant liver cancer cell lines [43].

Pathway analyses indicated a difference in inflammatory response between the two primary tumours, with upregulation of *OAS2*, *CCL2*, and *AKAP13* in Patient 1 compared to Patient 2 across all cell populations. Analysis of further tumours will be required to determine whether there is any consistent association between the presence of inflammatory markers and clinical behaviour of the tumour.

When examining expression of ~ 50 insulin/glucose-related DEGs between the two patients, *SLC2A2* was strongly expressed in the primary tumour and metastasis of Patient 2. High expression of *SLC2A2* expression has also been reported in some human insulinomas [44]. *SLC2A2* encodes the GLUT2 glucose transporter, which does not rely on insulin for transport of glucose across cell membranes and is essential for glucose sensing and homeostasis in beta cells.

An additional difference in insulin-expressing cells between patients was the upregulated expression of *CD40LG* in Patient 2. The encoded protein is typically expressed on the surface of T-cells and interacts with CD40 on the surface of B cells. Critically, *CD40* expression was one of the distinguishing markers of the B cells infiltrating the tumour microenvironment, and the cell communication analysis highlighted the *CD40LG* (*INS*^+^ *FOS*^*low*^, *INS*^+^) − CD40 (B cell) interaction. *CD40LG* plays a role in angiogenesis [1, 9] and has been described as a core prognostic indicator in breast cancer [50]. Soluble CD40L has been proposed as a promising biomarker in cancer diagnosis [32]. Therefore, *CD40L* deserves further attention in canine insulinoma.

Finally, this study provided an opportunity for single cell interrogation of a tumour and metastasis within the same patient (Patient 2). Unexpectedly, the metastasis showed significant > 20–70 fold upregulation in both insulin-expressing cancer cell populations of several marker genes typically associated with the exocrine pancreas, including *CLPS*, *ENSCAFG00000014481* (*PRSS2*), *ENSCAFG00000003818* (*PRSS*), and *CTRC*. The significance of this is not clear, but these genes might be important markers of metastatic transformation, invasion or associated with de-differentiation in canine insulinoma, in addition to their reported roles in pancreatitis [21, 22].

Functional pancreatic neuroendocrine tumours (pNETs) can be classified according to a range of features, including molecular markers, proliferative grading and hormone production [26]. Experimentally-induced pNETs in mice have previously been classified along GCG-INS axes [17]. Here, along similar GCG-INS axes, we have shown the insulin-expressing cells from the canine insulinoma samples examined show strong expression of INS and no expression of GCG, consistent with their clinical classification as insulinomas.

Human PanNETs have also been categorised at the whole-tumour level based on CNV patterns. Insulinomas lacked CNV deletions, unlike non-functional pancreatic neuroendocrine tumours, but CNV amplifications were observed. Tumours with CNV deletions had a high rate of loss-of-function mutations in tumour suppressor genes, and CNVs associated with elevated risk of relapse in non-functional tumours [16]. This contrasts with the canine insulinoma samples where whole-chromosome amplifications and deletions were detected in subsets of cells. Specifically, *INS*^+^ *FOS*^*low*^ cells, which have lost expression of key tumour suppressor genes, had more CNAs than the *INS*^+^ population. Likewise, more CNAs were detected in Patient 2, together suggesting that the acquisition of CNAs may be a marker of a more advanced disease state.

### Limitations of the study

The very small number of samples in this study, and the inclusion of insulinomas from only one breed of dog caveat generalising conclusions. The characterisation of transcriptomes in tumour-infiltrating immune cells would have had added value from comparison to matched peripheral blood samples. However, sufficient quantities of blood were not available after diagnostic use. Further hypotheses and observations on the tumour microenvironment and CNAs could be generated by comparison of the insulin-expressing cancer cells to the single cell transcriptome of healthy primary canine beta cells, but such data are not available at present.

### Future work

This study demonstrates the feasibility of acquiring single-cell data from veterinary surgical material that is surplus to diagnostic requirements. Future transcriptomic work will be of value using further samples and breeds and examining the impact of treatment with drugs in common use for insulinoma therapy, such as prednisolone and toceranib. In addition, larger sample numbers and matched tumour and blood whole genome sequencing data would allow analysis of the mutational landscape of canine insulinoma. This report represents the first description of the presence of more than one insulin-expressing cell type within spontaneous insulinoma. Spatial gene expression and epigenetic studies are also likely to reveal further important insights and help to identify treatment targets that may be shared across neuroendocrine tumours and species.

## Supplementary Information

Supplementary Material 1

Supplementary Material 2

Supplementary Material 3

Supplementary Material 4

Supplementary Material 5

Supplementary Material 6

## Data Availability

The scRNA-seq reads have been deposited in the European Nucleotide Archive (ENA project: PRJEB86016/study ERP169389; samples: ERS23781655, ERS23781656, and ERS23781657).
