# Supplementary material for: Single-cell transcriptomic analysis of canine insulinoma reveals distinct sub-populations of insulin-expressing cancer cells
Source: Vet Oncol. 2025 May 26;2(1):13. doi: 10.1186/s44356-025-00026-3 (PMC12106163; doi:10.1186/s44356-025-00026-3)
Supplement: Supplementary file 2 — Supplementary Material 2 [file 44356_2025_26_MOESM2_ESM.pdf]

Supplementary Figure 1

A.

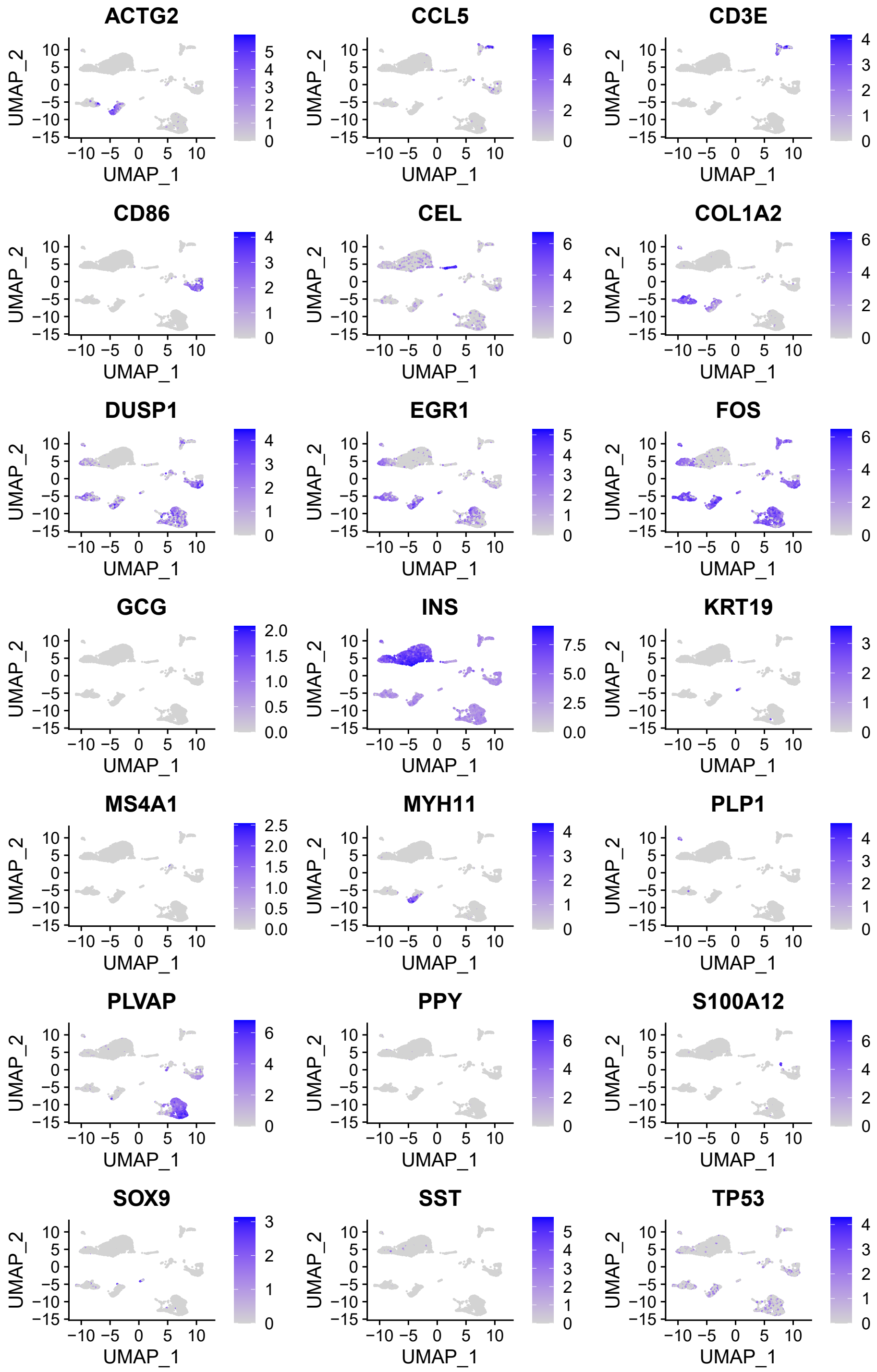

B.

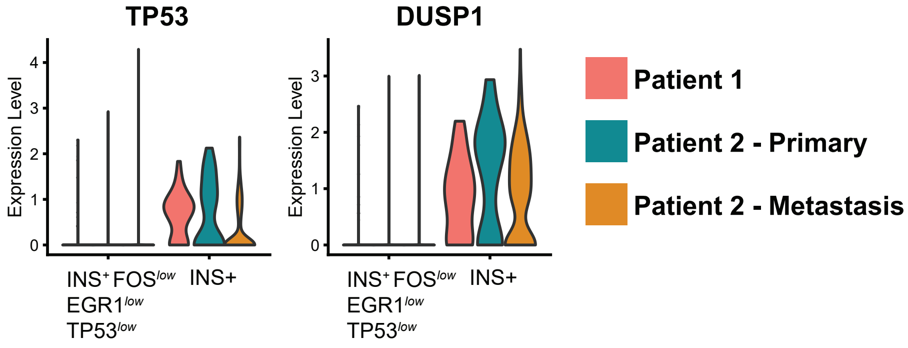

Supplementary Figure 1: Marker gene expression in canine insulinoma scRNA-seq. **A.** UMAP expression plots of standard marker genes and select markers distinguishing cell populations. **B.** Violin plots of TP53 & DUSP1 expression in insulin-expressing populations across samples.
