# Supplementary material for: Single-cell transcriptomic analysis of canine insulinoma reveals distinct sub-populations of insulin-expressing cancer cells
Source: Vet Oncol. 2025 May 26;2(1):13. doi: 10.1186/s44356-025-00026-3 (PMC12106163; doi:10.1186/s44356-025-00026-3)
Supplement: Supplementary file 3 — Supplementary Material 3 [file 44356_2025_26_MOESM3_ESM.pdf]

Supplementary Figure 2

A.

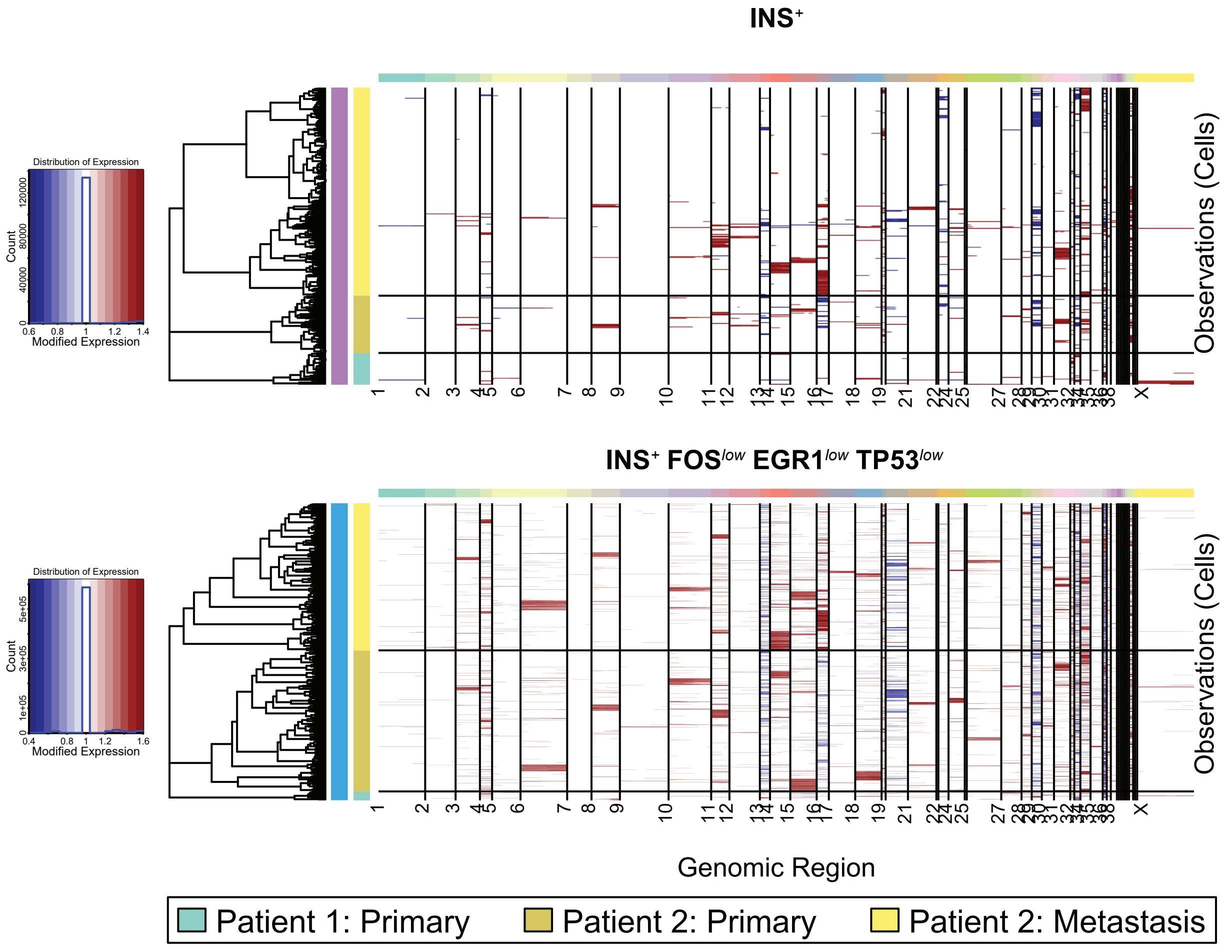

Supplementary Figure 2: Copy Number Alterations (CNAs) in canine insulinoma. **A.** Heatmap of CNAs in the INS^{+} (top) and INS^{+} FOS^{low} EGR1^{low} TP53^{low} (bottom) populations detected by InferCNV, where deletions are indicated in blue and amplifications are indicated in red.
