# Supplementary figures and images for: Single-cell transcriptomic analysis of canine insulinoma reveals distinct sub-populations of insulin-expressing cancer cells
Source: Vet Oncol. 2025 May 26;2(1):13. doi: 10.1186/s44356-025-00026-3 (PMC12106163; doi:10.1186/s44356-025-00026-3)

## Supplementary Figure 3

A.

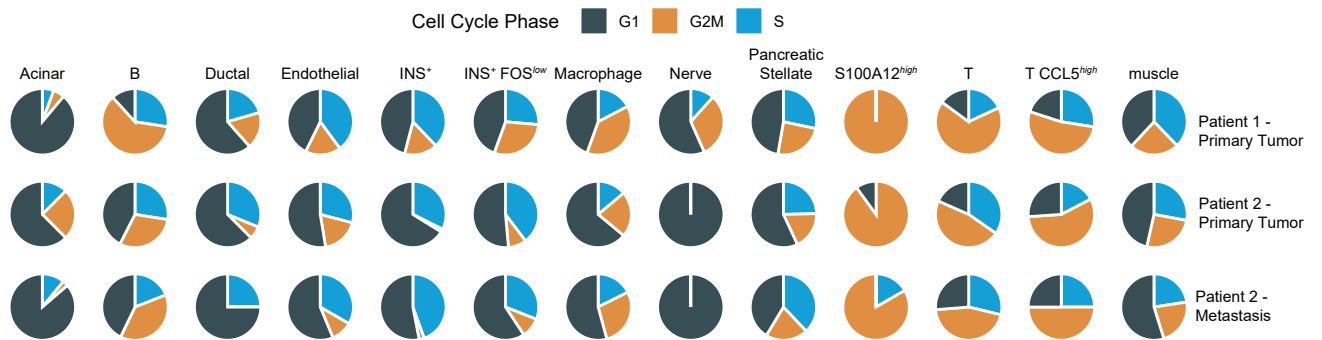

Supplementary Figure 3: Cell cycle phase by cell type and sample.

Supplement: Supplementary file 4 — Supplementary Material 4 [file 44356_2025_26_MOESM4_ESM.pdf]
