# Supplementary material for: Single-cell transcriptomic analysis of canine insulinoma reveals distinct sub-populations of insulin-expressing cancer cells
Source: Vet Oncol. 2025 May 26;2(1):13. doi: 10.1186/s44356-025-00026-3 (PMC12106163; doi:10.1186/s44356-025-00026-3)
Supplement: Supplementary file 5 — Supplementary Material 5 [file 44356_2025_26_MOESM5_ESM.pdf]

Supplementary Figure 4

A.

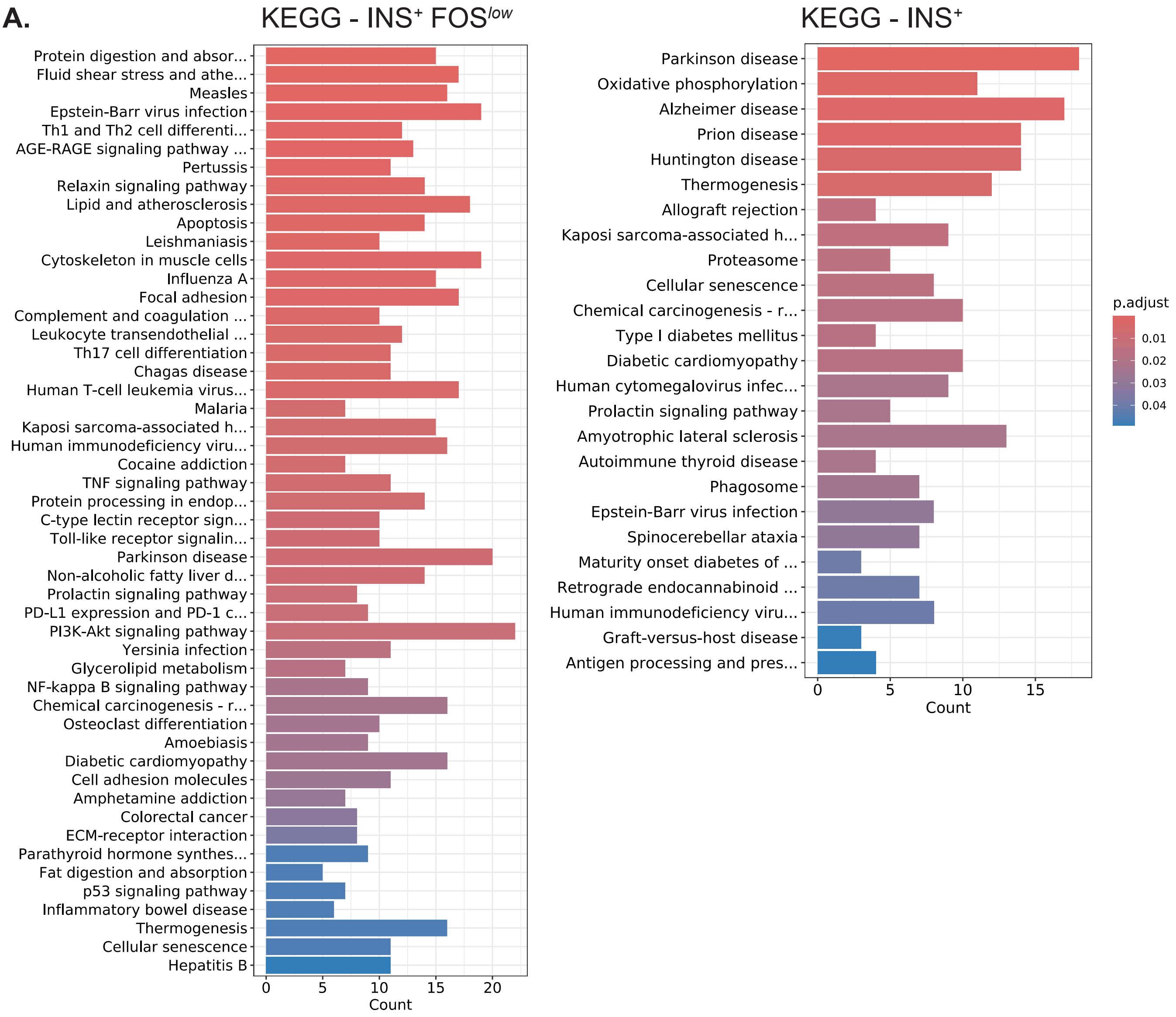

Supplementary Figure 4: Pathway analysis of DEGs between primary tumors in canine insulinoma
